# Effect of hirudin on serum matrix metalloproteinase-9 of acute cerebral infarction: A protocol of systematic review and meta-analysis

**DOI:** 10.1097/MD.0000000000020533

**Published:** 2020-07-02

**Authors:** Ying Bian, Ying Zhang, Zhi-bin Tian

**Affiliations:** a Department of Encephalopathy; b Department of Endocrinology, The Second Affiliated Hospital of Shaanxi University of Chinese Medicine; c Second Ward of Neurology Department, Xianyang Hospital of Yan’an University, Xianyang, China.

**Keywords:** acute cerebral infarction, effect, hirudin, matrix metalloproteinase-9, serum

## Abstract

**Background:**

This study aims to check the effect of hirudin on serum matrix metalloproteinase-9 (SMMP9) in patients with acute cerebral infarction (ACI).

**Methods:**

For acquisition of obtained data of included studies, we will undertake comprehensive search from the following electronic databases: MEDLINE, Embase, Cochrane Library, CINAHL, WANGFANG database, VIP database, CBM database, and China National Knowledge Infrastructure from their inceptions to the March 31, 2020. No restrictions of language and publication status will be applied to all database sources. Two investigators will independently undertake study selection, data extraction, and study quality. Any different opinions between 2 investigators will be solved by a third investigator through consultation. Study quality will be assessed using Cochrane risk of bias tool, and level of evidence for outcome results will be identified using the Grading of Recommendations Assessment, Development, and Evaluation method. We will use RevMan 5.3 software for statistical analysis.

**Results:**

From this study, we will evaluate the effect of hirudin on SMMP9 in patients with ACI.

**Conclusion:**

The findings of this study will provide evidence to ensure the effect of hirudin on SMMP9 in patients with ACI.

## 1 Introduction

Acute cerebral infarction (ACI), known as acute ischemic stroke, is a severe neurologic disorder.^[1–3]^ Published studies have reported that its high mortality and disability rates seriously impact quality of life in ACI patients.^[4–9]^ Thus, it is very important to treat ACI effectively and timely. Previous studies reported the association between serum matrix metalloproteinase-9 (SMMP9) in patients with ACI.^[10–12]^ Despite several studies have explored the effect of hirudin on SMMP9 in patients with ACI,^[13–25]^ no systematic review has evaluated the effect of hirudin on SMMP9 in ACI. Thus, the aim of this study is to assess the effect of hirudin on SMMP9 in patients with ACI.

## 2 Methods

### 2.1 Study registration

This study protocol has been registered in the INPLASY202040143. It is reported based on the Preferred Reporting Items for Systematic Review and Meta-Analysis Protocols statement.^[26]^

### 2.2 Eligibility criteria

#### 2.2.1 Type of studies

Only case-controlled studies (CCSs) will be included in this study. Other studies, such as animal studies, case studies, reviews, and non-CCSs will be excluded. There are no limitations on language and publication status.

#### 2.2.2 Type of participants

We will include CCSs on participants who are diagnosed as ACI. The race, gender, age, severity, and duration of CCSs are not restricted.

#### 2.2.3 Type of interventions

We will include studies using hirudin in patients with ACI in the experimental group.

We will consider studies using any management in patients with ACI in the control group.

#### 2.2.4 Type of outcomes

The primary outcome is SMMP9, as measured by enzyme-linked immunoassay kit or other methods.

The secondary outcomes are plasma positive pentamer protein 3, tissue plasminogen activator, high-sensitivity C-reactive protein level, hemorheology index, thromboplastin time, plasma prothrombin time, plasminogen activator inhibitor, thromboxane B2, improvement in neurologic deficits (as checked by simple intelligence status check scale, or other scales), and quality of life (as assessed by activities of daily living).

### 2.3 Search strategy

We will perform electronic search in the following electronic databases from their inceptions to the March 31, 2020: MEDLINE, Embase, Cochrane Library, CINAHL, WANGFANG database, VIP database, CBM database, and China National Knowledge Infrastructure. No limitations will be imposed on language and publication status. The sample of search strategy of MEDLINE is demonstrated in Table 1. Similar search strategies of other electronic databases will be adapted and modified.

**Table 1:**
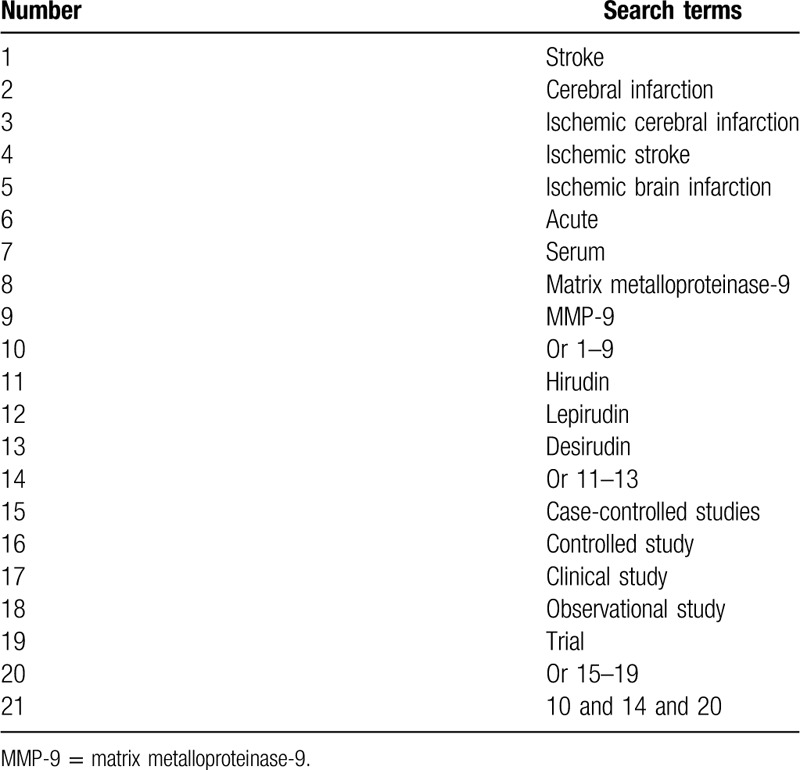
Search strategy for MEDLINE.

In addition, we will search conference proceedings, clinical trial registries, and reference lists of included studies.

### 2.4 Study selection

Two investigators will independently scan the titles and abstracts of all searched literature in accordance with the inclusion and exclusion criteria. When these literatures are unclear to judge whether they should be excluded, full-texts will be read to identity if they are qualified to be included. Any divergences between 2 investigators will be solved through discussion with another investigator. We will note reasons for all excluded studies. The flow chart will be used to present the whole process of study selection.

### 2.5 Data extraction

Two investigators will independently carry out data extraction, respectively. Any disagreements regarding the data extraction between 2 investigators will be resolved by another investigator. We will extract the following information: basic characteristics (including first author, time of publication, region, age and sex of patients, diagnostic criteria, inclusion, and exclusion criteria), study methods, details of managements (including types of interventions and controls, dosage, frequency, etc), all primary and secondary outcomes, and any other relevant information. If we identify some unclear or missing data, primary corresponding authors will be contacted to obtain it.

### 2.6 Study quality assessment

The methodologic quality of all included studies will be assessed by 2 independent investigators using Cochrane Risk of Bias Tool. It consists of 7 domains, and the judgments on each item is categorized as low risk of bias, unclear risk of bias, and high risk of bias. Any disagreements between 2 investigators will be solved by negotiation or consultation with the help of another investigator.

### 2.7 Statistical analysis

#### 2.7.1 Data synthesis

RevMan 5.3 software is used for statistical analysis. Mean difference or standardized mean difference and 95% confidence intervals will be used for expressing quantitative data, and risk ratio and 95% confidence intervals will be utilized for exerting dichotomous data. We will apply *I*^2^ statistic test to check statistical heterogeneity across studies. If *I*^2^ ≤ 50%, it means reasonable heterogeneity. If *I*^2^ > 50%, it suggests substantial heterogeneity. When *I*^2^ ≤ 50%, we will use a fixed-effects model. Otherwise, a random-effects model will be utilized. If sufficient data are obtained from at least 2 included studies, we will pool the data and will perform meta-analysis if reasonable heterogeneity is identified. If not, we will undertake subgroup analysis to check the factors for substantial heterogeneity.

#### 2.7.2 Subgroup analysis

We will explore the subgroup analysis according to on the different basic characteristics, study quality, interventions, controls, and outcome measurements.

#### 2.7.3 Sensitivity analysis

We will undertake sensitivity analysis to check robustness of merged results by removing low quality studies.

#### 2.7.4 Reporting bias

We will check reporting bias using Funnel plot and Egger regression test if at least 10 eligible studies are included.^[27,28]^

### 2.8 Grading the quality of evidence

We will identify evidence quality using the Grading of Recommendations Assessment, Development, and Evaluation method.^[29]^ It consists of 4 levels, and it is generally graded according to the basis of risk of bias, inconsistency, indirectness, inaccuracy, and publication bias.

### 2.9 Ethics and dissemination

No ethic approval is needed, because all data used in this study is collected from previous published literatures. We expect to publish this study on a peer-reviewed journal.

## 3 Discussion

The ACI is a very common neurologic disease, especially among the elderly population.

Although a wide range of treatments are available for ACI, there are still some shortcomings. Thus, it is still very necessary to explore more potential medications for this condition. Several studies have reported the effect of hirudin for the management of ACI, especially affects SMMP9 in patients with ACI. Unfortunately, no published systematic review has addressed this issue. Thus, this study will firstly explore the effect of hirudin on SMMP9 in patients with ACI. The results of this study expect to provide beneficial evidence for clinical practice and researchers.

## Author contributions

**Conceptualization:** Ying Bian, Ying Zhang, Zhi-bin Tian.

**Data curation:** Ying Bian, Ying Zhang, Zhi-bin Tian.

**Formal analysis:** Ying Bian, Ying Zhang, Zhi-bin Tian.

**Funding acquisition:** Zhi-bin Tian.

**Investigation:** Zhi-bin Tian.

**Methodology:** Ying Bian, Ying Zhang.

**Project administration:** Zhi-bin Tian.

**Resources:** Ying Bian, Ying Zhang.

**Software:** Ying Bian, Ying Zhang.

**Supervision:** Zhi-bin Tian.

**Validation:** Ying Bian, Ying Zhang, Zhi-bin Tian.

**Visualization:** Ying Bian, Ying Zhang, Zhi-bin Tian.

**Writing – original draft:** Ying Bian, Ying Zhang, Zhi-bin Tian.

**Writing – review & editing:** Ying Bian, Zhi-bin Tian.
